# Masturbation in Girls

**Published:** 1914-01

**Authors:** 


Masturbation in Girls. Diagnosis by Yeast Test. Kaufmann, *N. Y. Med. Jour.*, Oct. 18, 1913, makes a preliminary test of the urine to insure absence of yeast cells. In the evening, the child is given yeast to handle and put to bed without washing the hands. If yeast is found in the morning, it is considered diagnostic of masturbation. (If the domestic sterilization of the urine containers has been perfect and if yeast has not entered the urine from the anus, and if it has not subsequently entered the sample of urine from the air, and if the yeast drying on the hands has not been disseminated through the air beneath the bed covers, and if the nightgown or towel or handkerchief or sheet touched by the hands has not come in contact with the genitals, and if there has been no itching or other irritation of the parts leading to touch during sleep, without masturbation and, perhaps, some more ifs.—Ed.)

